# A Zero-Knowledge Proof-Enabled Blockchain-Based Academic Record Verification System

**DOI:** 10.3390/s25113450

**Published:** 2025-05-30

**Authors:** Juan Alamrio Berrios Moya, John Ayoade, Md Ashraf Uddin

**Affiliations:** 1 School of Engineering, Auckland University of Technology, 55 Wellesley Street East, Auckland Central, Auckland 1010, New Zealand; wtm7791@autuni.ac.nz; 2 School of Information Technology, Crown Institute of Higher Education, 116 Pacific Highway, North Sydney, NSW 2060, Australia; ashraf.uddin@cihe.edu.au

**Keywords:** academic credential fraud, blockchain, interplanetary file systems, security and privacy

## Abstract

Academic credential fraud presents a significant challenge to the global academic and labor markets, undermining the credibility of legitimate qualifications. In this paper, we introduce ZKBAR-V, a Zero-Knowledge Proof-Enabled Blockchain-Based Academic Record Verification System. This system is designed to provide a privacy-preserving, immutable, and secure framework for managing academic credentials. The proposed system leverages zkEVM smart contracts on a blockchain-based infrastructure that enables credential verification without exposing underlying data. The approach integrates Decentralized Identifiers (DIDs) to standardize identity management while eliminating reliance on centralized authorities. We have used dual-blockchain, which separates public and private information, which can enhance both efficiency and privacy. In addition, this approach employs the Interplanetary File System (IPFS) for decentralized and secure document storage. ZKBAR-V is designed as an open-source, interoperable solution with a standardized Application Programming Interface (API) for seamless integration. We implemented the system and conducted comprehensive testing, which demonstrates its capability to manage transactions securely, maintain privacy, and reduce costs compared to traditional Ethereum mainnet-based solutions. By combining advanced blockchain technologies, decentralized storage, and globally unique identifiers, ZKBAR-V offers a scalable, adaptable, and robust solution for academic credential management. This strategy can significantly enhance credential integrity, promote global student mobility, and provide institutions worldwide with a trustworthy and efficient verification system.

## 1. Introduction

Academic credential fraud has become a widespread global issue, contributing to a multi-billion-dollar black market for forged degrees and certificates [1]. The ease of accessing falsified credentials and the rising cost of education create an incentive structure that is difficult to regulate [2]. This fraudulent ecosystem not only devalues legitimate qualifications but also erodes trust in educational institutions, employers, and regulatory bodies [3]. The consequences extend beyond individual hiring decisions, impacting workforce competency, public safety, and economic stability. As academic institutions undergo digital transformation, they must navigate three critical challenges:

- Credential fraud—Ensuring that issued degrees are authentic and tamper-proof.
- Data privacy—Maintaining compliance with regulations like GDPR and FERPA while securing student information.
- Global interoperability—Creating a standardized system that allows credentials to be verified seamlessly across institutions, employers, and borders.

The increasing prevalence of fake academic credentials has become a critical challenge in the education sector, raising concerns about the integrity of academic qualifications. Traditional verification methods—such as physical certificates, emailed transcripts, and direct institutional communication—are often manual, time-consuming, and susceptible to forgery or manipulation, making it difficult for institutions and employers to authenticate credentials reliably [1]. As a result, there is a growing interest in blockchain technology as a solution to enhance the security, transparency, and interoperability of academic records. Blockchain-based credentialing systems provide tamper-proof, decentralized verification mechanisms, ensuring the authenticity and credibility of academic qualifications across institutions and geographical boundaries [4].

To address these challenges, various blockchain-based models have been proposed and developed, including Blockcerts [5], Hyperledger-based systems, and Ethereum-based credential verification frameworks. However, despite the potential benefits, there are still significant hurdles, such as scalability, privacy concerns, and regulatory compliance (e.g., GDPR). While some systems, such as B-ACVS and Blockcerts, have been successfully implemented, many remain at the theoretical stage with no fully functional prototypes. Moreover, existing solutions often lack interoperability, making it difficult to establish a global standard for academic credential verification. Research suggests that blockchain-based credential verification can enhance transparency, security, and efficiency in academic record management [6,7,8]. However, several challenges hinder widespread adoption:

- Scalability issues due to high gas fees and transaction costs on public blockchains.
- Lack of privacy mechanisms in existing blockchain solutions, exposing sensitive academic data.
- Interoperability barriers caused by differences in institutional infrastructure and digital identity management.

To overcome these limitations, we propose a ZKBAR-V (Zero-Knowledge Proof-Enabled Blockchain-Based Academic Record Verification) system, a comprehensive, privacy-preserving, and interoperable framework for managing academic credentials. ZKBAR-V is designed to enhance trust, security, and efficiency in academic verification processes by leveraging the following innovations:

- Zero-Knowledge Proofs (ZKPs)—Enables credential verification without revealing underlying student data [9,10], improving privacy while maintaining trust.
- Decentralized Identifiers (DIDs)—Establishes standardized, self-sovereign digital identities for students, facilitating global academic mobility without reliance on centralized identity systems [11].
- Dual-Blockchain Architecture (Public and Private Chains)—Separates publicly verifiable credential data from sensitive academic records, ensuring efficient, secure, and privacy-preserving transactions.
- Interplanetary File System (IPFS)—Provides secure, decentralized storage for academic documents, ensuring data integrity and tamper-proof verification in a distributed environment [12].
- zk-SNARK Encryption in zkEVM Smart Contracts—Implements privacy-preserving smart contracts, allowing credentials to be validated without exposing student information to third parties.

ZKBAR-V is built upon the Blockchain Academic Credential Interoperability Protocol (BACIP), which establishes ethical, legal, and technical standards for academic credential management [13]. The open-source nature of the system enables scalability, affordability, and global adoption, ensuring a low-cost, high-performance distributed architecture for secure, privacy-enhanced, and universally verifiable academic credentialing. Our article proposes the ZKBAR-V framework, which leverages zkEVM smart contracts, dual-blockchain architecture (public and private), decentralized identity (DID), and IPFS storage to securely manage and verify academic credentials. Unlike traditional centralized verification methods, ZKBAR-V enhances privacy and security using Zero-Knowledge Proofs (ZKPs), allowing credential verification without exposing sensitive data. Our main contributions are as follows:

- Privacy-Preserving Verification via ZKPs: We incorporate Zero-Knowledge Proofs to allow institutions and employers to verify the authenticity of academic credentials without revealing sensitive student data, offering stronger privacy than any existing system.
- Support for Self-Sovereign Identity with DIDs: ZKBAR-V leverages Decentralized Identifiers (DIDs) to provide globally interoperable, self-sovereign identities that eliminate the dependency on centralized databases for student identity verification.
- Dual-Blockchain Architecture: We design a hybrid architecture that separates public and private data across two blockchains, balancing transparency (public chain) with privacy and performance (private chain).
- IPFS Integration for Secure Document Storage: Academic records are stored off-chain using IPFS, ensuring decentralized, tamper-proof, and cost-effective data management.
- zk-SNARK-Enabled Smart Contracts on zkEVM: By deploying smart contracts on a zkEVM-compatible environment, ZKBAR-V allows for on-chain credential validation while maintaining full privacy and compliance with regulations such as GDPR.
- A Unified Framework for Global Interoperability: Unlike fragmented existing systems, ZKBAR-V is designed as a comprehensive and extensible platform that facilitates international recognition and seamless verification of credentials.

The remainder of this paper is organized as follows: Section 2 discusses related work. Section 3 describes the proposed framework, including its architecture. Section 4 presents the experimental setup and results, followed by a detailed performance evaluation. Finally, Section 6 concludes the paper and outlines future research directions.

## 2. Literature Review

In this section, we present several related works in this domain to illustrate the current state-of-the-art blockchain-based academic record management systems. Following this, we provide the background research that is relevant to our study.

Similar to our ZKBAR-V approach, some complex frameworks have been developed; however, many are still in the theoretical stage and do not have a fully functional prototype. For example, CredenceLedger is a theoretical study proposing a permissioned blockchain system for secure academic credential verification. While it outlines the framework, no prototype has been developed or tested [14]. Similarly, Badr et al. [15] proposed a permissioned blockchain-based system using Hyperledger to automate and secure the verification and transfer of academic records between institutions. While their paper outlines the system architecture, it remains theoretical, and no prototype has been developed or tested. Gaikwad et al. [16] also proposed a blockchain-based verification system for academic certificates that uses Ethereum, OCR, and smart contracts to automate the process of verifying academic credentials. The foundations for implementation are well established, but they did not materialize into a functional development. On the other hand, some systems have already been successfully implemented, allowing current use. For example, Nadeem et al. [17] developed a system called the Hybrid Blockchain-based Academic Credential Verification System (B-ACVS), which complies with GDPR regulations using a public blockchain based on Ethereum. In addition to the development, they implemented a functional system that was tested and validated.

MIT’s Blockcerts system [5,18] remains one of the most influential blockchain-based credentialing platforms, offering an open-source implementation that allows institutions to issue verifiable digital certificates on the Ethereum blockchain. However, it has limitations that constrain its applicability in privacy-sensitive or regulatory contexts. Notably, Blockcerts lacks native support for Zero-Knowledge Proofs, which may potentially cause verification processes to expose sensitive metadata. Furthermore, its initial design did not include Decentralized Identifiers (DIDs), thereby limiting self-sovereign identity capabilities. Its reliance on a single-layer public blockchain also makes it less adaptable to institutions that need to separate public attestations from confidential academic data. In contrast, ZKBAR-V enhances these foundational features by incorporating ZKPs, DIDs, and a dual-chain model, enabling scalable, privacy-preserving, and regulation-compliant academic verification across institutional and national boundaries. On the other hand, Sony Global Education [19] created a framework for credential registration and accreditation, but without using a public platform like Ethereum. Instead, it uses a private network supported by Hyperledger Fabric deployed using IBM Cloud. This was because it did not need to be a decentralized system, but rather, this is a system designed to manage foreign students arriving in Japan, with a variety of languages and learning paths. In this way, they can have an unforgeable academic verification system. Even though this system is fully developed and functionally active, its source codes are not openly available; in fact, it is a system that operates only privately in Japan. Learning Machine, who collaborated with MIT Media Lab in the creation of Blockcerts, was acquired by Hyland Credentials, who then created a paid version of this system using Blockcerts as a base by changing some components and offering more support and infrastructure for the integration of the system, allowing institutions to delegate responsibilities; but this system is not open source. This platform does not have IPFS integrated, as it was integrated into Blockcerts after its collaboration with Learning Machine; however, Hyland did add Decentralized Identifiers [20].

There have been several other solutions developed to secure the verification and usage of certificates in universities and higher education institutions around the globe. Due to the ease of obtaining fake credentials, institutions are increasingly investing in technologies that distribute academic credentials in a secure and immutable way to ensure authenticity and reduce fraud.

Education plays a vital role in societal development, but the rise of fake certificates is a growing concern—from paper-based forgeries to database tampering. To address this, a blockchain-based overlay mechanism was proposed for securely storing and verifying genuine digital certificates [21]. The system ensures immutability, transparency, and tamper-proof verification, allowing certificates to remain permanently accessible for reference. A working prototype developed on the Ethereum test network confirmed its security and feasibility for credential management. Expanding on this, Rahman et al. [4] presented a blockchain-IPFS-based model targeting Southeast Asia, where the prevalence of fake certificates undermines employment opportunities. Their approach combines temporary databases with IPFS to generate hash codes stored on blockchain nodes, enabling employers to verify credentials cost-effectively while maintaining trust in academic records.

In another regional context, Alnafrah et al. [22] proposed a national hybrid blockchain platform designed for low-income countries, such as Syria and Sudan. Their system connects students, universities, governments, and employers to securely manage academic records. Tested successfully in both countries, the platform enhances education accessibility and aligns with the Sustainable Development Goal 4, promoting lifelong learning and international mobility. To improve certificate revocation and maintain trust in issued credentials, Baldi et al. [23] developed a blockchain-based revocation mechanism using public ledgers. While it mitigated single points of failure, users were required to manually query Certificate Authorities to check revocation lists, which proved inefficient.

While prior blockchain-based credential systems, such as that proposed by Baldi et al. [23], recognize the potential benefits of decentralization, they also face vulnerabilities to blockchain-specific threats—such as 51% attacks, Sybil nodes, or Denial of Service vectors—particularly when relying on open, public networks. In our ZKBAR-V, we address these concerns by adopting a dual-chain strategy that separates public verification from private academic operations. The private blockchain component operates in a permissioned environment, reducing susceptibility to external tampering. In addition, identity verification and credential issuance are controlled via DIDs and regulated through secure smart contracts. Our use of zk-SNARK-based verification ensures minimal on-chain data exposure and resistance to manipulation, thus enhancing the overall trust model of our system. Lei et al. [24] attempted to address these inefficiencies by integrating revocation within security and privacy protocols. However, their framework faced scalability challenges and was vulnerable to blockchain-based attacks. Building on the need for efficient infrastructure, Haveri et al. [25] developed an Ethereum-based multi-node private blockchain system with off-chain document storage via IPFS. Despite enhanced data decentralization, the use of Proof-of-Work resulted in high resource consumption. In [24], the reliance on a PoW-based blockchain introduces high energy consumption and longer transaction finality times, which can be barriers to real-time academic verification at scale. While this could be mitigated by adopting a different consensus algorithm, such a change would require a redesign of the system’s trust model and infrastructure. Pawar et al., using the same system, noted high CPU usage and low system performance due to similar architectural demands. While [25] demonstrates a practical blockchain implementation using Ethereum and IPFS, it reports high CPU usage, which we believe may be a result of non-optimized smart contract logic and on-chain computation overhead. Rather than critiquing the resource usage in isolation, we highlight this to contrast with ZKBAR-V’s off-chain zk-proof computation and dual-chain design, which separates sensitive processing from public verification, improving both efficiency and privacy. In traditional systems like the one described by Sethia [26], verifying whether a credential has been revoked typically involves querying a centralized or off-chain revocation list. While this method works, it can introduce delays and reduce the system’s decentralization, especially when the revocation list must be regularly synchronized or manually checked by relying parties. In ZKBAR-V, we address this limitation by integrating revocation control within the smart contract layer itself. Each credential has an associated status flag on-chain, which can be toggled by authorized issuers. During verification, the system checks the credential’s current status as part of the Zero-Knowledge Proof verification flow, ensuring that only valid, non-revoked credentials are accepted, without requiring off-chain dependencies.

To support standardization and cross-platform integration, the Blockchain Academic Credential Interoperability Protocol (BACIP) was introduced by Berrios Moya [13]. BACIP addresses the lack of global interoperability by offering a framework that facilitates issuance, storage, and verification of academic credentials across borders. It leverages blockchain’s core benefits—immutability and decentralization—to enhance trust, particularly in cross-border verification scenarios.

Further exploring interoperability and secure frameworks, Shaikh et al. [27] proposed a Hyperledger Sawtooth-based consortium model for academic credentialing. The system uses smart contracts to automate registration, issuance, and storage of credentials, while integrating machine learning and SHA-256 encryption to prevent tampering. Tested on benchmark educational datasets, the model demonstrated high accuracy and tamper resistance. Chowdhary et al. [28] introduced a secure credential system that links blockchain-stored certificates to students using unique identifiers and secret phrases. Verified through government-issued identity proofs, this system ensured integrity of both personal and credential data while offering secure issuance and reception of academic documents. Similarly, Delgado et al. [29] proposed a regulatory-compliant model to issue and verify academic credentials using blockchain. Their model places particular emphasis on user privacy and system scalability, aiming to pave the way for a globally accepted academic certification solution. Responding to the need for digital transformation in education post-COVID-19, Deenmahomed et al. [30] designed a blockchain-based system for managing exams, transcripts, and certificates. Their lightweight architecture prioritizes transparency, anonymity, and data availability, showing strong potential to modernize academic credentialing in a decentralized format.

Although early systems such as the University of Nicosia’s Bitcoin-based credentialing [31] introduced the idea of blockchain for academic verification, their reliance on Bitcoin imposes constraints including lack of smart contract support and poor scalability. MIT’s Blockcerts [18], while widely adopted, does not incorporate privacy-preserving mechanisms like ZKPs and initially lacked support for Decentralized Identifiers (DIDs), which were only introduced later in derivatives such as Hyland Credentials [20]. Other solutions like B-ACVS [17] and VeriFi [4] utilize Ethereum and IPFS for secure storage but offer limited privacy control and operate primarily on public chains, which can expose metadata and raise compliance issues with data protection regulations. Moreover, most systems lack a unified approach to global interoperability. ZKBAR-V addresses these challenges by integrating a dual-chain architecture to separate sensitive and verifiable data, using zk-SNARKs within zkEVM smart contracts for privacy-preserving validation, and embedding DID support for decentralized identity control, thus offering a more robust and scalable framework for global academic credential verification.

The proposed ZKBAR-V introduces several innovations that collectively distinguish it from existing solutions in the academic credential verification landscape. While many prior systems, such as Blockcerts, B-ACVS, and VeriFi, utilize blockchain and IPFS for secure storage and verification, they often fall short in ensuring full privacy, interoperability, or self-sovereign identity. ZKBAR-V addresses these gaps by incorporating Zero-Knowledge Proofs (ZKPs) to verify credentials without disclosing personal student data, thereby offering a significant advancement in privacy protection—something absent in all the compared models. Moreover, while some systems adopt Decentralized Identifiers (DIDs) or dual-blockchain configurations, none integrate all three core innovations: DIDs, a dual-blockchain architecture (public and private chain separation), and zk-SNARK-enabled smart contracts in a zkEVM environment. This integration allows ZKBAR-V to maintain GDPR compliance while enabling scalable, cross-border academic verification. In contrast to systems like Hyland Credentials or BACIP, which support parts of this functionality, ZKBAR-V combines privacy, decentralization, and interoperability into a unified, secure framework, making it a pioneering step toward global, tamper-proof, and privacy-preserving academic record management. We present a comparison of our approach with existing state-of-the-art systems in Table 1. In the table, ZKP refers to Zero-Knowledge Proofs, DID stands for Decentralized Identifiers, IPFS denotes the Interplanetary File System, and zk-SNARK or zkEVM represents the use of Zero-Knowledge Succinct Non-Interactive Arguments of Knowledge within a zkEVM-compatible smart contract environment.

While systems such as those proposed by Lei et al. [24] and Haveri et al. [25] are not included in Table 1, they are discussed in the literature review due to their technical relevance. Specifically, Lei et al. contributed insights into revocation mechanisms and blockchain security vulnerabilities, while Haveri et al. explored resource-intensive smart contract designs and off-chain storage integration. However, these systems do not offer full academic credential management pipelines or lack several of the key dimensions we used in our comparison, such as privacy-preserving verification, dual-chain design, or interoperability features like DIDs. Similarly, systems like those from Chowdhary et al. [28], Delgado et al. [29], and Deenmahomed et al. [30] are acknowledged for their focused innovations (e.g., identity linking, compliance models, and post-COVID credential digitization), but are not comprehensive frameworks, which is why they are omitted from the feature comparison table. Instead, our comparison table emphasizes end-to-end systems that directly align with ZKBAR-V’s goals: secure issuance, privacy-preserving verification, revocation handling, and global interoperability.

### Background Study

Blockchain technology was introduced by Nakamoto [32] in 2008, who proposed a decentralized, secure, and transparent model for information distribution and transaction processing. This technology operates through a network of nodes that verify and validate the authenticity of stored information using ledgers organized into cryptographically linked blocks, forming an immutable chain. Initially implemented in Bitcoin, blockchain has since been widely adopted across various sectors, including finance, supply chain management, and education, in both private and public networks:

- Public Blockchain: Bitcoin is a prime example of a blockchain operating on a public network—a fully decentralized system that functions without a central governing entity. In this model, every participant can validate transactions using a consensus mechanism such as Proof of Work or Proof of Stake, which ensures network security by requiring validator nodes to verify transactions. While this system provides a highly secure and trust-based structure for transactions, it faces challenges in scalability and high operational costs due to the extensive validation process required by multiple participants [10]. Nevertheless, for applications where security and data immutability are paramount, public blockchains remain one of the most effective solutions.
- Private Blockchain: Unlike public blockchains, private blockchains operate within a restricted network while maintaining the core principles of security and immutability. However, they lack full decentralization, as control is centralized under the entity that implements and manages the system. Private blockchains are typically used at the enterprise level, where the primary focus is on data security rather than decentralization. In these networks, only a selected group of participants have permission to conduct transactions and validate data. One key advantage of private blockchains is the absence of gas fees, unlike public networks such as Ethereum. Instead, the primary costs are related to implementation and administration [33].
- The Zero-Knowledge Ethereum Virtual Machine (zkEVM) integrates Zero-Knowledge Proofs (zk-SNARKs or zk-STARKs) with smart contract execution on Ethereum-compatible networks. This technology enables transaction validation without exposing sensitive data, significantly enhancing privacy in blockchain networks. zkEVM can be deployed on both public and private networks. In public blockchains like Ethereum, it ensures transaction privacy while maintaining network security. In private environments, zkEVM provides a scalable and confidential solution for businesses requiring secure transactions. According to [9], Zero-Knowledge Proofs are essential for privacy in blockchain applications, allowing verification without disclosing underlying data. In addition, recent research highlights zkEVM’s compatibility with Solidity-based smart contracts, facilitating seamless integration across various blockchain networks while enhancing security and scalability [9,10].
- Decentralized Storage: The Interplanetary File System (IPFS) is a decentralized file storage system that operates without reliance on a central server, similar in principle to blockchain but designed for handling large volumes of data, such as files. In the IPFS, files are broken into smaller chunks and distributed across a network of computers, known as nodes. This redundancy ensures that even if some nodes go offline, the files remain accessible. Unlike traditional storage systems where files are retrieved by name or location, IPFS uses content-addressable storage. Each file is identified by a unique cryptographic hash rather than a filename. If a file is modified, its hash changes, making any tampering immediately detectable. This mechanism enables the detection and removal of corrupted files while preserving the integrity of the network [12]. A powerful use case arises when IPFS is combined with blockchain technology. Blockchain ensures transaction integrity and verification, while IPFS provides efficient decentralized storage for larger, unstructured data that blockchain alone cannot handle efficiently. This integration enhances security, scalability, and reliability while maintaining decentralized principles [12].
- Decentralized Identifiers (DIDs): Decentralized Identifiers (DIDs) were developed to address key challenges in identity registration and management, particularly the centralization of identity systems controlled by governments or traditional institutions. In centralized systems, individuals lack full control over their personal data after registration, making their information susceptible to security breaches and unauthorized access. Beyond security concerns, portability issues arise due to variations in identity formats across different countries. Some nations use distinct naming conventions or lack standardized identity numbers, making cross-border identity verification complex. Verifying identities outside the jurisdiction of the issuing government requires an in-depth understanding of each country’s registration structure, further complicating the process. To solve these challenges, the World Wide Web Consortium (W3C) formed the DID Working Group in 2017 to develop a decentralized identity standard. In 2020, they released DID v1.0, incorporating key attributes such as decentralization, user control, privacy, security, proof-based verification, discoverability, interoperability, portability, simplicity, and extensibility [11]. The ZKBAR-V framework integrates DIDs as a core component to align with BACIP principles, enhancing system interoperability through a standardized global identification system. This approach enables credential verification without relying on centralized authorities, ensuring compliance with international regulations such as GDPR, which emphasize data security and privacy.

## 3. The Proposed Zero-Knowledge-Based Academic Record Verification System

The architecture of the ZKBAR-V framework provided a secure decentralized environment to store the academic credentials in a privacy-preserving manner and make the credentials interoperable with other systems adhering to the standards outlined in the Blockchain Academic Credential Interoperability Protocol (BACIP). In this framework, we utilize two blockchains: individual private blockchain for institutions and a public blockchain for all parties including institutions, students, third-party stakeholders like job recruiters. Every institution maintains their private blockchain for storing student’s academic record in an encrypted format. The roles of the two zkEVM-based blockchains in securing academic documents and credentials are described below.

- Private zkEVM:
  – This handles credential issuance and stores sensitive academic data (personally identifiable information such as social data).
  – The institution stores security number, passport, age, academic result, and misconducts on the private blockchain.
  – The institution also uses IPFS for decentralized encrypted document storage.
- Public zkEVM
  – Public zkEVM generates Zero-Knowledge Proofs (zk-Proofs) without exposing private data.
  – This processes credential verification by validating zk-Proofs.
  – It stores only credential hashes on the public blockchain (ensuring privacy).
  – It manages revocation proofs, updating the public blockchain when credentials are revoked.

**Figure 1:**
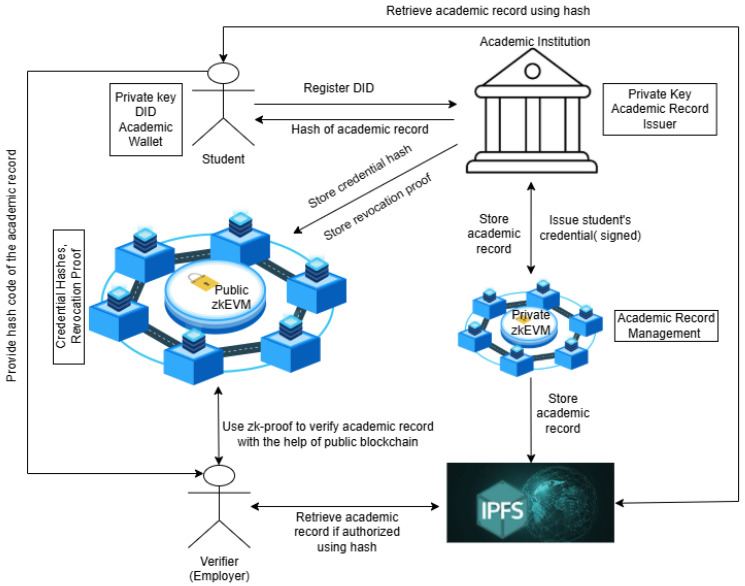
Zero-Knowledge -based record verification system.

In this proposed framework, each student possesses a wallet that contains their private key, public key, and other credentials like hash of the academic records. The student’s public key is considered as the address of their blockchain. Students utilize the private key to sign and control access to their academic records. During registration, the student generates a Decentralized Identifier (DID) on the private blockchain, which is owned by an Institution. The DID is linked with the student’s institution and public key. The academic institution, using its private key, verifies the student’s identity and records the data in the private blockchain for ensuring secure and tamper-proof credential management. The framework of zero-knowledge based academic record verification system is presented in Figure 1.

Once the student’s identity is verified, the institution issues academic credentials containing details such as degree information, grades (if available), and course specifics. These credentials are stored securely on the private blockchain and the Interplanetary File System (IPFS) through zkEVM-based smart contracts. When a student completes a course, the institution digitally signs the credential using its private key and securely stores it in IPFS and the private blockchain.

To enhance privacy and security, a Zero-Knowledge Proof (ZKP) is generated, ensuring that only a credential hash is recorded on the public blockchain for verification. The student then receives a verifiable credential linked to their DID, which is stored in their academic wallet. When verification is required (e.g., by an employer or another academic institution), the student shares the ZKP, allowing the verifier to query the public blockchain to validate the credential hash. The verifier confirms the credential’s authenticity using the public keys of both the student and the issuing institution. Since zkEVM enables Zero-Knowledge verification, the verifier can authenticate the credential without accessing the actual document. If additional verification is needed, the verifier can request access to the private blockchain via zkEVM smart contracts. In cases where a credential must be revoked due to fraud, misconduct, or errors, the institution updates the private blockchain and it generates a revocation proof that is then stored on the public blockchain. This allows verifiers to check the revocation status, ensuring the integrity of academic records. Since students cannot modify or remove revoked credentials, the system maintains trust and authenticity. If a student earns a new degree or requires an update to their credentials, the institution issues a new digitally signed credential on the private blockchain and public blockchain, ensuring seamless academic record management.

With credentials stored via DIDs and zkEVM-based smart contracts, students can securely share verified academic records across institutions and job markets without exposing sensitive information. Institutions and employers can validate credentials efficiently without requiring direct access to documents, ensuring both privacy and interoperability. In addition, governments and accreditation bodies can monitor and detect fraudulent records through blockchain transparency, reinforcing trust and security within the academic ecosystem.

### 3.1. System Actors and Roles

In the previous section, we mention the different actors for this framework. Table 2 summarizes the actors and their roles in this academic record management system.

### 3.2. Security and Privacy Requirement

A student applies for a job, and the employer needs to verify their degree. The student shares a zk-Proof linked to his DID. The employer queries the public blockchain for credential validity. If needed, the verifier requests permission to access additional verification details via the private blockchain. The verification process confirms authenticity without revealing raw academic records, ensuring privacy. The security and privacy requirement are summarized in Table 3.

### 3.3. Zero-Knowledge Proof Implementation

Zero-Knowledge Proofs (ZKPs) are cryptographic protocols that allow one party (the prover) to convince another party (the verifier) that a statement is true, without revealing the underlying data that support the statement. In the context of ZKBAR-V, this means a student can prove their possession of a valid academic credential—such as a degree issued by a recognized institution—without disclosing the full credential or personal information. Specifically, we use zk-SNARKs (Zero-Knowledge Succinct Non-Interactive Arguments of Knowledge), which are compact and efficient. The student generates a zk-SNARK proof off-chain, asserting that their credential meets certain conditions (e.g., issued by Institution X, degree = “MSc”, status = “valid”), and submits this proof to a smart contract on a zkEVM-compatible blockchain. The smart contract then verifies the proof’s validity without accessing the actual credential. This enables privacy-preserving verification and helps comply with data protection regulations, such as GDPR, by ensuring that personal data are never revealed or stored on-chain.

### 3.4. Threat Model for the Proposed System

The STRIDE model presented in Table 4 and Table 5 categorizes threats into spoofing, tampering, repudiation, information disclosure, Denial of Service, and elevation of privilege. Below is a structured threat model for the framework, detailing threat actors, threats, and mitigation strategies.

**Figure 2:**
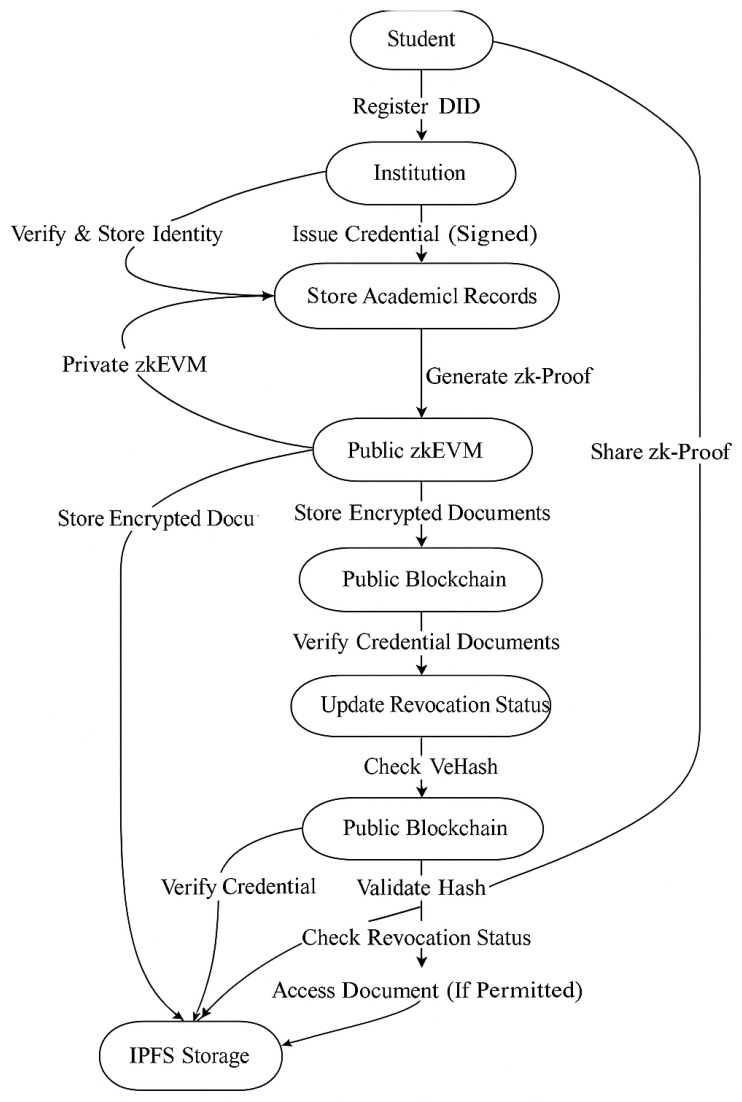
Workflow of Zero-Knowledge-based record verification system.

Table 6 presents the classification of data that can be stored in the private and public blockchain to ensure privacy and security of every actor in the framework. Some use cases of the proposed academic record verification system are discussed below. Figure 2 presents the workflow of the proposed academic record verification system.

The ZKBAR-V framework leverages blockchain-based security mechanisms, including Zero-Knowledge Proofs (ZKPs), Decentralized Identifiers (DIDs), and zkEVM-based smart contracts to ensure data integrity, privacy, and authentication. However, despite these security features, potential risks remain in different areas of the system. The DREAD risk assessment has been conducted to evaluate various threats systematically, taking into account the inherent security benefits of blockchain. We assess a subjective risk using the DREAD risk model, which is presented in Table 7. The DREAD model scores threats based on the following parameters:

- D (Damage Potential)—The severity of the impact if the threat is exploited.
- R (Reproducibility)—How easily the threat can be reproduced.
- E (Exploitability)—The ease with which the threat can be exploited.
- A (Affected Users)—The number of users impacted by the threat.
- D (Discoverability)—How easy it is for an attacker to discover the vulnerability.

The overall Risk Score is calculated as follows: $RiskScore=\frac{D+R+E+A+D}{5}$, where each factor is rated on a scale from 1 (low) to 10 (high).

**Table 7:** DREAD risk assessment.

Component	Threat	D	R	E	A	D	Risk Score	Severity
Private zkEVM	Spoofing (Unauthorized Access)	4	3	3	4	3	3.4	Low
	Tampering (Modifying Records)	5	2	2	5	2	3.2	Low
	Information Disclosure	5	3	3	6	3	4.0	Medium
	Denial of Service (DoS)	3	2	2	4	2	2.6	Low
	Elevation of Privilege	6	3	3	5	3	4.0	Medium
Public zkEVM	Spoofing (Impersonation)	3	3	3	4	3	3.2	Low
	Tampering (Hash Manipulation)	4	2	2	4	2	2.8	Low
	Repudiation (Credential Denial)	3	2	2	4	2	2.6	Low
	Denial of Service (Overloading Network)	5	3	3	5	3	3.8	Medium
IPFS	Tampering (Modifying Files)	4	2	2	5	2	3.0	Low
	Information Disclosure	6	3	3	6	3	4.2	Medium
	Denial of Service (Access Disruption)	4	3	3	4	3	3.4	Low
DID and ZKP Processes	Spoofing (Fake Identity)	3	2	2	4	2	2.6	Low
	Information Disclosure	5	3	3	6	3	4.0	Medium
	Repudiation (Denying Credential Issuance)	3	2	2	4	2	2.6	Low

The ZKBAR-V model indicates low risk for tampering and spoofing due to its utilization of blockchain-based authentication procedures. The use of cryptographic signatures makes identity spoofing virtually impossible since transactions and ownership of credentials can be traced on the blockchain. The inability of blockchain records to be accessed for tampering with credentials stored is a certainty that tampering is not possible, hence the assurance of a high degree of data integrity. However, information disclosure remains a medium risk primarily due to potential private key mismanagement. While all sensitive data are encrypted, the security of academic records relies on how securely users handle their cryptographic keys. Moreover, while IPFS provides decentralized encrypted storage, there remains a risk of metadata leakage, which could disclose sensitive information even if the content is encrypted. In Denial of Service (DoS) attacks, the threat level ranges from low to medium based on the nature of the blockchain. Private blockchains are less vulnerable to DoS attacks as they are permissioned and only allow access to trusted parties. But public zkEVM blockchains that can support credential verification are still prone to DoS attacks. Nonetheless, the distributed nature of blockchain networks minimizes this threat because transactions are disseminated across nodes, and flooding the system becomes challenging.

Lastly, privilege elevation attacks are of medium severity if smart contracts are not securely implemented. If access control policies are also configured inappropriately, attackers can exploit permission loopholes to gain access to unauthorized resources or execute privileged actions. To avoid such attacks, the framework must incorporate strong access control mechanisms that will only allow authorized entities to carry out specific actions. Utilizing multi-signature approvals for sensitive actions can also reduce the likelihood of such attacks.

## 4. Results and Discussion

The ZKBAR-V framework was developed by selecting relevant blockchain-based scholarly credential management systems obtained from the reliable academic literature, such as Blockcerts, Sony Global Education, or Hyland Credentials. The ZKBAR-V system was compared with existing systems based on the following criteria: the selected type of blockchain (public, private, or hybrid); applied privacy mechanisms (e.g., Zero-Knowledge Proofs); applied data management strategies (e.g., the use of IPFS for data decentralized storage); applied identity management strategies (e.g., Decentralized Identifiers); and applied data protection regulations (e.g., GDPR). These data were collected from the documentation of the systems, academic papers, and public reports. For each system, architecture, privacy, data management, and legal compliance were analyzed to enable a clear distinction between ZKBAR-V and other solutions in terms of architecture, technical features, and legal compliance.

### 4.1. Development and Implementation

**Figure 3:**
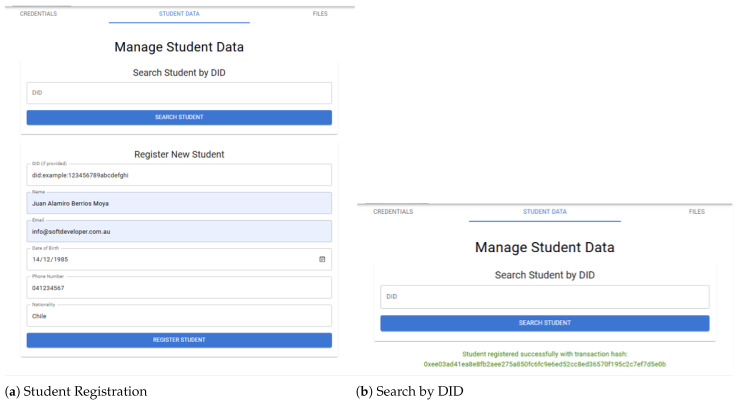
The GUI for a students’ wallet.

The development and implementation of ZKBAR-V followed an iterative approach. The system was set up in a way that it is functional, secure and interoperable. The core of the data structure was built using Solidity to develop smart contracts for issuance, verification, and revocation of credentials, which were tested locally using Ganache and Truffle before deploying the contracts on the public zkEVM blockchain. The wallet of front-end presented in Figure 3, built using React.js, provided a user-friendly experience to the students and the institutions so that they could interact with the system, while the back-end, built using Node.js, handled the blockchain side and took care of storing data on IPFS. Communication and interaction between the front-end and back-end were ensured through the well-designed API. The front-end and the back-end of the system processed the interaction between the students and the institutions through the IPFS data structure to ensure that the integrity and the verifiability of the system were maintained. The IPFS layer was used to store huge academic documents so that they could be decentralized, and the integrity of the documents was maintained through cryptographic hashes recorded on various nodes on the blockchain. The system was tested and validated to ensure the smooth deployment and the operational efficiency of all the components.

The ZKBAR-V framework was successfully developed, resulting in a fully functional system for decentralized academic credential management. Key components such as smart contracts, APIs, and front-end/back-end integrations were implemented and thoroughly tested. The smart contracts, written in Solidity, handle essential tasks such as credential issuance, verification, and revocation on the public zkEVM blockchain, while sensitive data are securely managed on a private zkEVM network. Both networks were designed to ensure security, privacy, and interoperability.

A significant outcome of this phase was the release of the ZKBAR-V codebase and documentation under an open-source license. The repository, which includes the smart contracts, API documentation, installation instructions, and IPFS integration details, is available on github.com/portfoliojuanberrios/ZKBAR-V. The open-source license encourages collaboration and adoption, allowing academic institutions and developers to freely implement, modify, and improve the system. This approach promotes innovation while reducing the technical and financial barriers to decentralized credential management, aligning with the project’s mission of global standardization and transparency.

### 4.2. Comparison of Component of the Proposed System with Existing Systems

Analyzing the current systems that have a practical implementation of frameworks for the management of academic credentials using blockchain.

The ZKBAR-V framework presented Figure 1 is based on the distribution of credentials through a public zkEVM network, but also integrates a private zkEVM for managing data that do not need to be shared.

In this way, ZKBAR-V is the only one to propose a dual system that also uses the same EVM technology. Unlike other systems that decide to use one or the other platform, this allows ZKBAR-V to efficiently distribute data by sharing only the strictly necessary ones on the public network. ZKBAR-V, along with Hyland Credentials, are the only ones that have integrated the Decentralized Identity system, allowing for the standardization of identity management on a global scale, in addition to increasing students’ control over the verification of their personal data. An IPFS system that allows for storing files as unstructured data in a secure and decentralized manner, such as certificates documents, passport pictures, or others, in various formats like PDFs, images, etc., has been adopted by ZKBAR-V, Blockcerts, and Sony Global Education. Regarding complying with GDPR standards, all these systems stated that they follow these rules in their frameworks.

Table 8 shows the presence of various operational components based on blockchain supporting the management of academic credentials. Figure 2 shows how ZKBAR-V integrates most components such as IPFS, decentralization, public blockchain, GDPR, and private blockchain.

### 4.3. Validation and Testing

**Figure 4:**
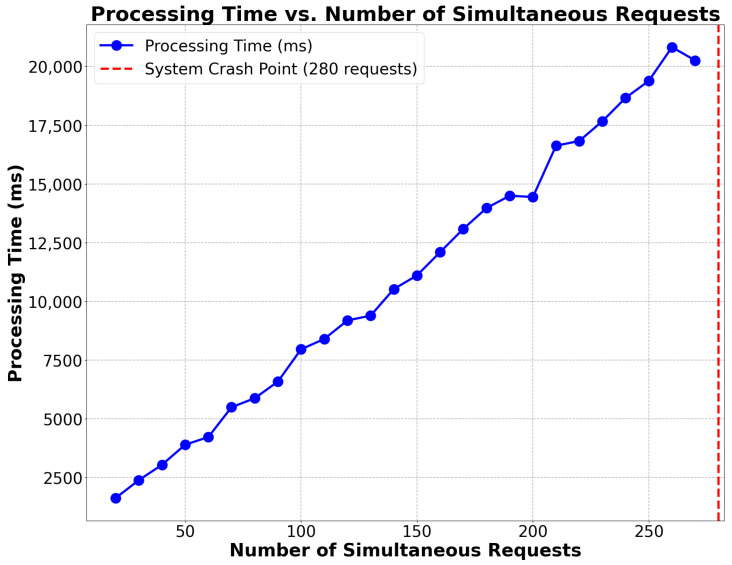
Processing time vs. number of simultaneous requests.

**Figure 5:**
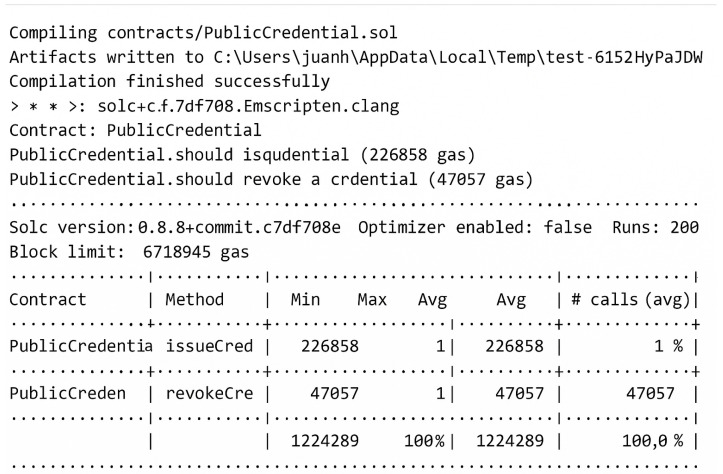
Gas required for smart contract of the proposed system.

Testing of the ZKBAR-V framework was conducted across several domains to ensure that the system could operate at scale under various conditions and within the constraints of security and compliance. The testing process was designed to capture important aspects of system scalability, security, integrity of data, functionality and cost:

- Scalability Testing: This test was performed to determine how the system performs with increasing numbers of concurrent credential issuance/verification requests. The test started with lower loads and incrementally increased the number of concurrent requests, measuring processing time, resource utilization (CPU and memory), and response times to find a threshold, for instance, at the point where we can expect the system to slow down in terms of performance. The test helped us determine the system’s maximum capacity before a threshold is exceeded.
  The Scalability Testing revealed that the ZKBAR-V framework was capable of efficiently managing up to 280 concurrent credential issuance and verification requests without significant performance issues. During this load, resource utilization, including CPU and memory usage, remained within acceptable limits, and response times were consistent. However, when the load surpassed 280 concurrent requests, there was a marked increase in processing time and resource consumption, leading to noticeable delays in response times. CPU usage peaked significantly, and the system began experiencing bottlenecks, indicating that its operational capacity had been reached. These results highlight the system’s robustness at moderate traffic levels but suggest that optimizations are necessary to support higher volumes without performance degradation.
  Figure 4 shows a graph illustrating the relationship between the time required to process a certain number of simultaneous requests and to mark the breaking point of the system’s collapse.
- Data Integrity and Privacy Testing: The Data Integrity and Privacy Testing of academic credentials managed by the ZKBAR-V framework was carried out in a zkEVM environment. The process involved uploading a batch of academic documents to IPFS and generating their respective SHA-256 cryptographic hashes, which were then recorded in the zkEVM blockchain. In the first part of the testing, the documents were retrieved from IPFS and verified against the stored hashes. If any unauthorized changes were detected, it indicated that the system was able to prevent any tampering with documents. In the second part of the testing, the same batch of verifications was carried out, but this time using Zero-Knowledge Proofs (zk-SNARKs). Although the documents were verified, no adjacent data (including personal information) could be exposed in this part of the testing. The tests monitored both the system’s ability to maintain document integrity, as well as its ability to keep the system private at the time of credential verification.
  The Data Integrity and Privacy Testing on the zkEVM environment confirmed that the ZKBAR-V framework was still robust and uncompromised. The cryptographic hashes of the documents (stored on the zkEVM blockchain and linked via a hash to the data on IPFS) were tested for consistency. The system proved impervious to any kind of trickery in document updates, as all documents that were not changed produced matching hashes. Modifications to documents resulted in mismatched hashes, as did attempts to create hash collisions. All these malicious-intent scenarios were blocked at their source. Privacy tests also confirmed that the zk-SNARKs implemented in the ZKBAR-V framework were operating well and allowed the system to authenticate credentials (e.g., university qualifications) without revealing other, adjacent personal data. The zkEVM network sent out only cryptographic proofs, and the data themselves remained fully protected. This confirmed that ZKBAR-V does indeed offer a strong extra layer of privacy. The system is GDPR-compliant.
- Functional Testing: This ensured that the system functioned as a whole, meaning from end-to-end. We tested credential issuance, verification, and revocation, and how the front-end (user interface) interacted with the back-end (blockchain interactions) and IPFS. The functional testing verified that smart contracts executed as expected and that the data handling functions functioned as intended so the system would work as expected under normal usage.
  The integration and interplay of all the elements of the ZKBAR-V framework for Functional Testing was successful. This included the front-end, back-end, smart contracts, and integration with external systems. The system worked smoothly when issuing credentials, verifying credentials, and for revoking credentials, all performed correctly using the smart contracts in the zkEVM blockchain. The front-end was built in React.js for a smooth interaction experience for the students and institutions, and the back.js actively listens to requisite information from the front-end, communicates with the blockchain, and securely stores the documents on IPFS. Sensitive data protection: Positive results considered as sensitive information were strictly protected for all transaction cycles. Unit tests (smart contracts and API): No errors or security vulnerabilities identified. Web app: All connections with external systems by API are functional; no errors. Functional tests: Operational for all transactions, all steps, and all conditions. The system operates properly, with no errors or security vulnerabilities discovered. The system is ready for use. We can feed external systems with information and use the web app.
- Cost Efficiency Analysis: The Cost Efficiency Analysis analyzed the cost effectiveness of issuing, verifying, and revoking credentials on the public zkEVM network. It tracked gas fees and other transaction costs as part of the system’s operation in the zkEVM. It also compared the costs of deploying ZKBAR-V in the zkEVM to deploying the system on Bitcoin and Ethereum. These simulations allowed us to gauge the performance of the zkEVM relative to other blockchain networks. We identified optimizations that would minimize transaction costs for the system while keeping it economically viable for academic institutions.
  The analysis, based on gas prices published on 9 September 2024 (Polygon, 2024; Etherscan, 2024) [34,35], demonstrated that zkEVM Polygon consistently provided approximately 94% cost savings across all operations of issuing a credential, revoking a credential, and contract deployment when compared to Ethereum Mainnet. These findings underscore the substantial cost advantage of using zkEVM Polygon for the decentralized management of academic credentials. Figure 4 illustrates the gas consumption for the ZKBAR-V system, while Figure 5 visualizes the cost differences between both networks for credential-related transactions. Moreover, by segregating sensitive data into a private network, ZKBAR-V minimizes public transaction costs, as only necessary credential issuance and verification data incur public network fees. This dual-network approach further enhances the system’s cost efficiency.
  Figure 5 Gas calculation of smart contract PublicCredential.sol on the ZKBAR-V System. Figure 5 shows the gas used for the PublicCredential.sol smart contract. It shows the gas consumed to issue (226,858 gas), revoke (47,057 gas), and deploy (1,224,829 gas).

**Figure 6:**
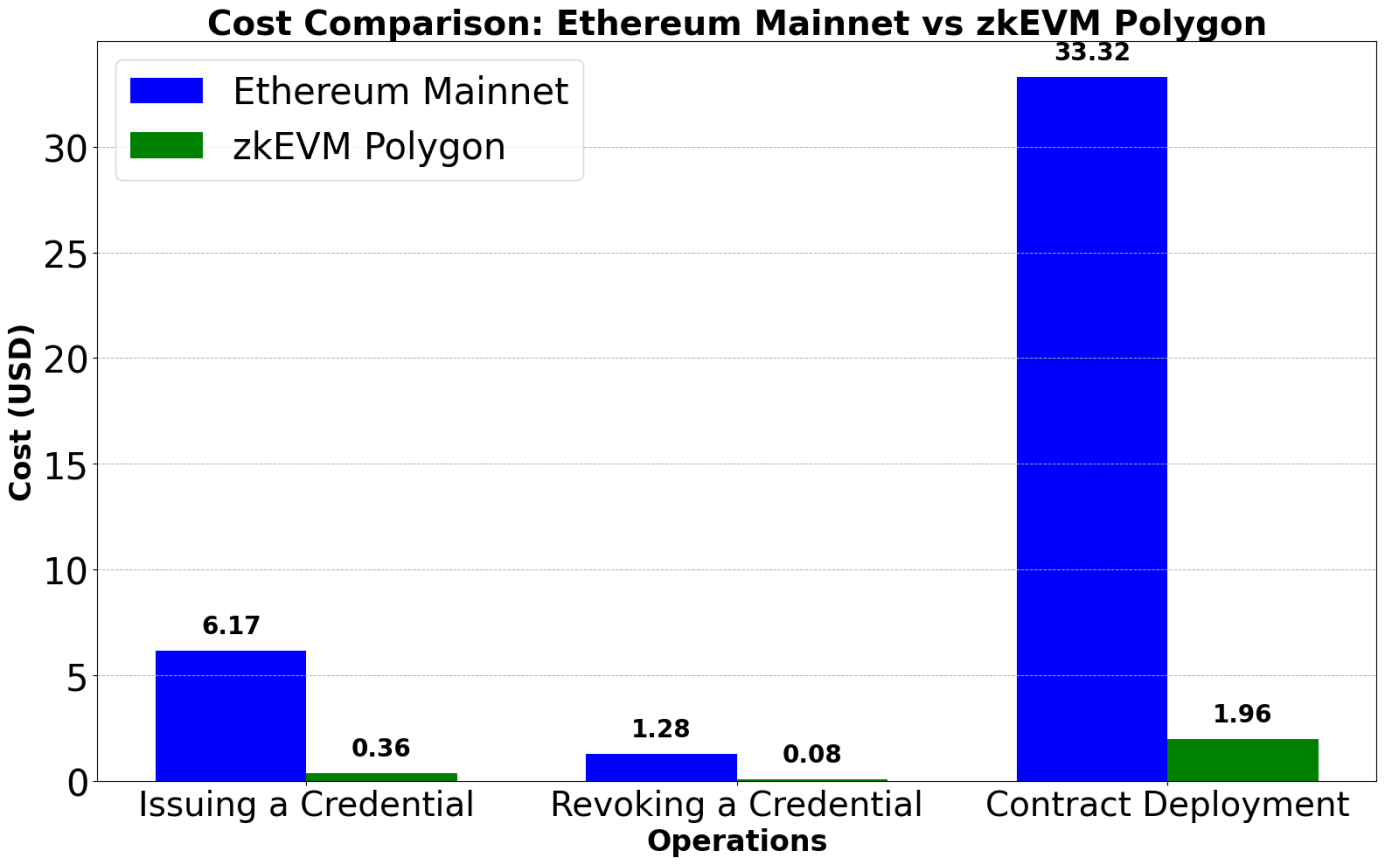
Cost comparison between Ethereum Mainnet and zkEVM Polygon of the proposed system.

Figure 6 Cost comparison between Ethereum Mainnet and zkEVM Polygon for credential issuance, revocation, and contract deployment in the ZKBAR-V system. This graph illustrates the cost efficiency differences between Ethereum Mainnet and zkEVM Polygon for issuing a credential, revoking a credential, and deploying the PublicCredential.sol smart contract. On average, zkEVM Polygon reduces transaction costs by approximately 94% compared to Ethereum Mainnet, as calculated based on gas prices and the operations related to academic credentials management.

**Figure 7:**
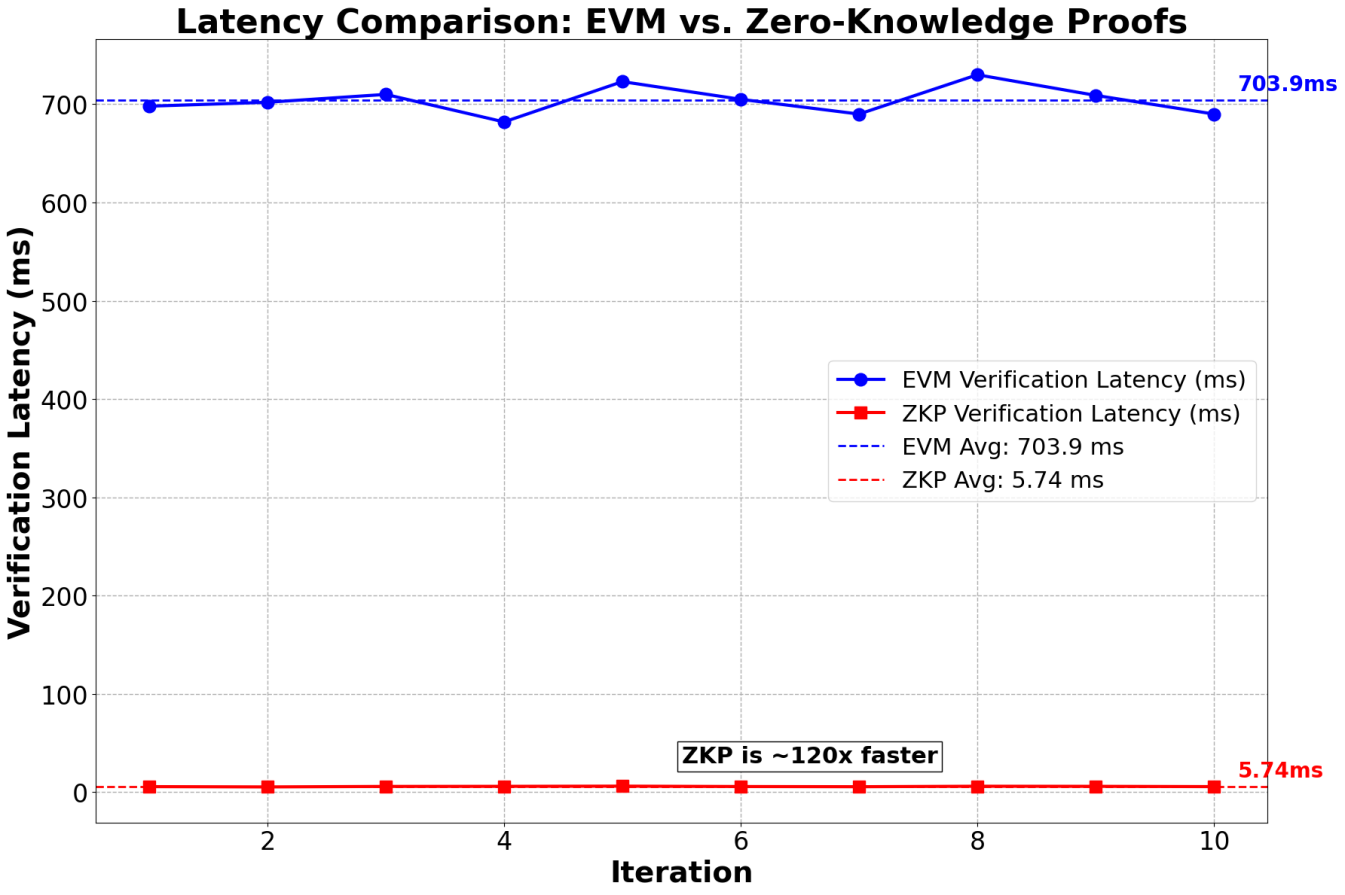
Latency comparison between Ethereum Virtual Machine vs. Zero-Knowledge Proofs for blockchain.

- Latency comparison between zkEVM and EVM: A verification latency comparison between Ethereum Virtual Machine (EVM) and Zero-Knowledge Proofs (ZKPs) was conducted over 10 iterations, measuring the time required to verify a transaction or computation result. The results presented in Figure 7 show that EVM has an average verification latency of 708 ms (±0.02 s), while ZKP takes only 5.8 ms (±0.001 s), meaning that ZKP is approximately 120 times faster, with significantly lower execution variability. This analysis confirms that ZKP is a more efficient solution for improving scalability and reducing costs in blockchain, as it enables fast and lightweight transaction validations without requiring full computation on every node import time.

## 5. Discussion

Although our system addresses several critical challenges—such as data privacy, revocation, and cross-border verification—it does not yet evaluate real-time IPFS retrieval performance, multi-language record support, or accessibility for users with low digital literacy. These are important factors for adoption, especially in low-resource environments. As part of future work, we plan to conduct large-scale pilot deployments, user studies, and performance benchmarking under varying network conditions.

### 5.1. Usability of the Proposed System

To ensure that the proposed ZKBAR-V system can be effectively adopted by institutions, students, and credential verifiers, we designed the user experience around simplicity and accessibility. The system provides web-based dashboards for institutions to issue and manage credentials, a student-facing mobile interface for managing personal credentials and consent-based access control, and a verifier portal that supports instant credential validation via QR code or credential hash. All cryptographic operations, such as ZKP generation and on-chain validation, are abstracted from the user to minimize friction. By integrating revocation status and credential metadata into the interface, stakeholders can interact with the system without needing in-depth technical knowledge, thus improving usability and trust.

### 5.2. Adoption Challenges

While ZKBAR-V offers technical innovations for privacy-preserving, decentralized academic credential verification, real-world adoption may face non-technical hurdles. Institutional resistance is common due to legacy systems, budget constraints, or reluctance to adopt blockchain-based infrastructures. Regulatory concerns such as data protection laws (e.g., GDPR, FERPA) may require legal review of cross-border data flows and smart contract governance. Additionally, digital literacy gaps among students and university staff can hinder effective use, especially in developing countries or non-technical faculties. To mitigate these, future work should explore user training modules, legal framework alignment, and pilot deployments in collaboration with educational institutions and government bodies.

## 6. Conclusions and Future Work

The development and implementation of the ZKBAR-V framework mark a significant advancement in decentralized academic credential management. Leveraging the capabilities of both public and private zkEVM blockchains, ZKBAR-V ensures the integrity and privacy of academic records through robust cryptographic methods, including Zero-Knowledge Proofs (zk-SNARKs) and decentralized storage via IPFS. This dual-network architecture not only enhances data security and privacy but also achieves cost efficiency, as demonstrated by the approximately 94% reduction in operational costs when compared to Ethereum Mainnet. Through comprehensive testing—ranging from scalability and data integrity to functional performance and cost analysis—ZKBAR-V has proven to be a secure, scalable, and interoperable solution for academic institutions worldwide. By open-sourcing the framework, ZKBAR-V fosters global adoption and innovation, encouraging institutions to implement, modify, and improve the system. This aligns with the framework’s mission of establishing a global standard for the secure and decentralized verification of academic credentials, adhering to legal and privacy standards like GDPR. Ultimately, ZKBAR-V presents a future-proof solution to the challenges of credential fraud, data privacy, and interoperability, positioning itself as a leading framework in the realm of academic credential verification. Future work will include evaluating system-level performance metrics such as IPFS retrieval latency, response times under varying network loads, and real-world deployment scenarios to further validate the scalability and usability of the ZKBAR-V framework.

## Figures and Tables

**Table 1:** Comparison of blockchain credential systems.

System/Study	ZKP	DID	Dual-Chain	IPFS	zk-SNARK or zkEVM	Prototype
CredenceLedger [14]	No	No	No	No	No	No
Badr et al. [15]	No	No	No	No	No	No
Gaikwad et al. [16]	No	No	No	Yes	No	No
B-ACVS (Nadeem et al.) [17]	No	No	No	Yes	No	Yes
Blockcerts (MIT) [18]	No	No	No	Yes	No	Yes
Sony Global Education [19]	No	No	Yes (Private Only)	No	No	Yes
Hyland Credentials [20]	No	Yes	No	No	No	Yes
University of Nicosia [31]	No	No	No	No	No	Yes
BACIP (Moya) [13]	No	Yes	No	No	No	Unknown
Baldi et al. [23]	No	No	No	No	No	Yes
Lei et al. [24]	No	No	No	No	No	No
Shaikh et al. [27]	No	No	Yes	No	No	Yes
Chowdhary et al. [28]	No	Yes	No	No	No	Yes
Delgado et al. [29]	No	Yes	No	No	No	No
Deenmahomed et al. [30]	No	No	No	No	No	Yes
Rama et al. [21]	No	No	No	Yes	No	Yes
VeriFi (Rahman et al.) [4]	No	No	No	Yes	No	Yes
Alnafrah et al. [22]	No	Yes	Yes	No	No	Yes
ZKBAR-V (Our Approach)	Yes	Yes	Yes	Yes	Yes	Yes

**Table 2:** Actors and their roles in the system.

Actor	Role and Responsibilities
Student	Owns academic credentials. Registers Decentralized Identifiers (DIDs). Signs transactions. Shares Zero-Knowledge Proofs (zk-Proofs) with verifiers.
Academic Institution	Issues credentials. Verifies student identity. Digitally signs credentials. Revokes credentials when necessary.
Verifier (Employer/University)	Requests credential verification. Checks credential hashes. Uses Zero-Knowledge Proofs (zk-Proofs) to confirm authenticity.
zkEVM Smart Contracts	Executes credential issuance, verification, and revocation logic using Zero-Knowledge Proofs.
Public Blockchain	Stores credential hashes and revocation proofs for transparency and trust.
Private Blockchain	Stores sensitive academic records while ensuring privacy.
IPFS (Decentralized Storage)	Stores academic documents (e.g., transcripts) securely with cryptographic integrity.

**Table 3:** Security and privacy requirements.

Requirement	Description
Data Confidentiality	Sensitive academic records are stored in a private blockchain and not exposed publicly.
Zero-Knowledge Verification	Credential authenticity is verified without revealing actual records.
Immutability and Tamper Resistance	Credential hashes stored on the public blockchain prevent modification or forgery.
User Control and Access	Students control who accesses their credentials using Decentralized Identifiers (DIDs) and Zero-Knowledge Proofs (zk-Proofs).
Decentralization and Trust Minimization	No single entity can manipulate records, ensuring trustless verification.

**Table 4:** Threat actors and their capabilities.

Threat Actor	Description	Potential Actions
Malicious Student	A dishonest student attempting to forge credentials.	Attempts spoofing. Attempts tampering. Attempts repudiation.
Compromised Institution	A legitimate institution whose security is breached.	Engages in tampering. Engages in repudiation. Causes data leaks.
Unauthorized Verifier	A verifier (e.g., employer/university) that accesses credentials without consent.	Causes information disclosure. Engages in repudiation.
External Attacker	A hacker or fraudulent entity outside the system.	Engages in spoofing. Engages in tampering. Causes information disclosure. Performs Denial of Service. Attempts privilege escalation.

**Table 5:** STRIDE threat analysis of the proposed system.

STRIDE Threat	Description	Impact on the System	Mitigation Strategies
S—Spoofing	Impersonation of students, institutions, or verifiers.	Fake credentials, unauthorized access to verification.	Decentralized Identifiers (DIDs), public–private key authentication, Zero-Knowledge Proofs (zk-Proofs) for verification.
T—Tampering	Unauthorized modification of academic records.	Altered credentials, fake certifications.	Immutable blockchain storage, zk-SNARKs for credential verification.
R—Repudiation	Denying issuance or verification of credentials.	Institutions or students falsely claim actions were not performed.	Digital signatures on transactions, Zero-Knowledge Proofs (zk-Proofs) to verify transactions.
I—Information Disclosure	Unauthorized access to private academic data.	Privacy breaches, exposure of sensitive student records.	zkEVM smart contracts, data encryption, access control policies.
D—Denial of Service (DoS)	Overloading the system to disrupt credential verification.	Students or employers unable to verify credentials.	Rate-limiting, distributed storage (IPFS), and efficient blockchain queries.
E—Elevation of Privilege	Unauthorized access to private blockchain or issuing credentials.	A compromised institution can fraudulently issue degrees.	Permissioned private blockchain, institution key-based access control.

**Table 6:** Data privacy and public vs. private information.

Type of Data	Storage Location	Who Can Access?
Credential Hashes	Public Blockchain	Verifiers (Read-Only)
Revocation Proofs	Public Blockchain	Verifiers (Read-Only)
Full Credential Details	Private Blockchain	Student Institution
DID (Decentralized ID)	Private Blockchain	Student Institution Verifier (Limited)
IPFS Stored Transcripts	IPFS (Encrypted)	Only Authorized Verifiers (Decryption Key)

**Table 8:** Comparison of the proposed ZKBAR-V with other systems.

Components	ZKBAR-V	Hyland Credentials	B-ACVS	Sony Global Education	BCERT
GDPR	Yes	Yes	Yes	Yes	Yes
IPFS	Yes	No	No	No	Yes
Decentralized Identifiers (DIDs)	Yes	Yes	No	No	No
Private Blockchain (Hyperledger)	Yes	No	No	Yes	No
Public Blockchain (Ethereum)	Yes	Yes	Yes	No	Yes
Dual-Blockchain (Public zk-EVM and Private zk-EVM)	Yes	No	No	No	No

## Data Availability

The implementation code and supporting data are available in the following GitHub repository: https://github.com/portfoliojuanberrios/GARV-BI, accessed on 10 April 2025.
